# Imaging, biomarker and invasive assessment of diffuse left ventricular myocardial fibrosis in atrial fibrillation

**DOI:** 10.1186/s12968-020-0603-y

**Published:** 2020-02-10

**Authors:** Gordon A. Begg, Peter P. Swoboda, Rashed Karim, Tobias Oesterlein, Kawal Rhode, Arun V. Holden, John P. Greenwood, Eduard Shantsila, Gregory Y. H. Lip, Sven Plein, Muzahir H. Tayebjee

**Affiliations:** 1 Department of Cardiology, Leeds General Infirmary, X39 Cardiology Offices, Great George St, Leeds, LS1 3EX UK; 2 Leeds Institute of Cardiovascular and Metabolic Medicine, University of Leeds, Clarendon Way, Leeds, LS2 9JT UK; 3 Department of Biomedical Engineering, King’s College, London, UK; 4 Institute of Biomedical Engineering, Karlsruhe Institute of Technology (KIT), 76131 Karlsruhe, Germany; 5 MCRC and School of Biomedical Sciences, University of Leeds, Leeds, LS2 9JT UK; 6 University of Birmingham Institute of Cardiovascular Sciences, City Hospital, Birmingham, UK; 7 University of Liverpool, Liverpool, UK; 8 Aalborg Thrombosis Research Unit, Department of Clinical Medicine, Aalborg University, Aalborg, Denmark

**Keywords:** Atrial fibrillation, Cardiovascular magnetic resonance imaging, Fibrosis, Biomarkers, Voltage mapping

## Abstract

**Background:**

Using cardiovascular magnetic resonance imaging (CMR), it is possible to detect diffuse fibrosis of the left ventricle (LV) in patients with atrial fibrillation (AF), which may be independently associated with recurrence of AF after ablation. By conducting CMR, clinical, electrophysiology and biomarker assessment we planned to investigate LV myocardial fibrosis in patients undergoing AF ablation.

**Methods:**

LV fibrosis was assessed by T1 mapping in 31 patients undergoing percutaneous ablation for AF. Galectin-3, coronary sinus type I collagen C terminal telopeptide (ICTP), and type III procollagen N terminal peptide were measured with ELISA. Comparison was made between groups above and below the median for LV extracellular volume fraction (ECV), followed by regression analysis.

**Results:**

On linear regression analysis LV ECV had significant associations with invasive left atrial pressure (Beta 0.49, *P* = 0.008) and coronary sinus ICTP (Beta 0.75, *P* < 0.001), which remained significant on multivariable regression.

**Conclusion:**

LV fibrosis in patients with AF is associated with left atrial pressure and invasively measured levels of ICTP turnover biomarker.

## Background

Percutaneous pulmonary vein (PV) isolation is often used for rhythm control in patients with atrial fibrillation (AF). However, at least one third of such patients experience a recurrence of AF even after multiple procedures [1]. This is most commonly due to reconnection of the pulmonary veins, however in a significant proportion of patients this is not the case and the mechanism(s) in these instances is unclear. Identification of AF patients who are likely to maintain sinus rhythm after the procedure is important, to reduce unnecessary exposure to procedural risks and expense.

Fibrosis is a hallmark of the left atrial (LA) pathological changes associated with AF development and recurrence after ablation, and research has explored the clinical utility of LA fibrosis assessment by various methods [2–6].

However, left ventricular (LV) fibrosis is also more prominent in AF patients than those without AF, and may be a predictor of AF recurrence [7, 8]. Diffuse LV fibrosis can be estimated using cardiovascular magnetic resonance (CMR), by calculating the extracellular volume fraction (ECV) from native and post contrast T1 mapping [9].

Circulating biomarkers such as type I collagen C terminal telopeptide (ICTP), type III procollagen N terminal peptide (PIIINP) and galectin 3 (gal-3) are markers of fibrosis that can be measured in the bloodstream [2]. They offer minimally invasive assessment of fibrosis, and would be a useful tool for improving patient selection if their clinical utility in doing so could be confirmed. They may also have a research application, in defining the mechanism of AF.

Although LV fibrosis has been associated to some extent with AF and AF recurrence after treatment, the mechanism behind this association is not clear. Raised LA pressure has been associated with recurrence of AF after catheter ablation, however the relationship between LA pressure and ventricular cardiac fibrosis in AF patients has not been studied in depth [10]. LA pressure is a routinely available direct measurement during AF procedures after trans-septal puncture, and further study may provide mechanistic insights into any haemodynamic influence on LV fibrosis in this patient group.

We investigated the interaction between LV fibrosis, LA fibrosis, and LA pressure, all of which have been associated with arrhythmia recurrence in patients after AF ablation. This interaction was examined in a multi-modality fashion, using CMR, invasive LA voltage mapping, LA pressure measurement and circulating biomarker assays. We hypothesized that LA fibrosis, measured by voltage mapping, is associated with diffuse LV fibrosis, measured by T1 mapping, and that raised LA pressure is associated with both of these measures. To attempt to gain a mechanistic insight into the pathological process of the fibrosis identified via these imaging methods, we also tested levels of circulating fibrosis biomarkers, including from intracardiac blood.

## Methods

Ethical approval was granted by the UK National Research Ethics Service Committee - Leeds West (ref. 13/YH/0349). Thirty-one patients undergoing first-time LA ablation for paroxysmal, persistent, or long-standing-persistent AF were recruited at the Leeds General Infirmary between September 2014 and August 2015, as part of a wider study consecutive cohort (*n* = 93) undergoing biomarker assessment before ablation. Details of the wider cohort have been published [6, 11]. Of this cohort, 31 participants was the maximum number that could be recruited to the CMR study presented in this article. Patients with systemic inflammatory disease, recent or active malignancy, severe kidney disease (estimated glomerular filtration rate (eGFR) < 30 ml/min/1.73m^2^) connective tissue disease, or any contra-indication to CMR were excluded. Written informed consent was gained from all participants.

CMR scans were carried out on a dedicated 1.5 Tesla CMR scanner (Ingenia, Philips Healthcare, Best, Netherlands). Venepuncture was performed immediately prior to the scan, and blood was inserted into potassium EDTA tubes for on-site analysis of haematocrit on a ADVIA 2120 analyser (Siemens Healthineers, Erlangen, Germany). Cine imaging in multiple planes was performed, to allow measurement of standard LA and LV dimensions. Native T1 maps were acquired (electrocardiogram (ECG) triggered 5 s(3 s)3 s Modified Look Locker Inversion Recovery (MOLLI) scheme, reconstructed voxel size 1.2 × 1.2x10mm^3^) on a mid-ventricular short axis slice. Fifteen minutes after administration of 0.15 mmol/kg intravenous gadolinium based contrast agent a post-contrast T1 map was acquired with identical planning (4 s(2 s)3 s(2 s)2 s MOLLI). ECV was calculated from the pre and post contrast T1 maps [9].

Radiofrequency (RF) ablation was performed according to the 2012 international consensus statement [12]. Under conscious sedation or local anaesthetic, venous access was obtained via the right and left femoral veins. After trans-septal puncture, LA bipolar voltages were recorded using a high-density circular electrophysiological (EP) mapping catheter and 3D mapping system (Lasso/CARTO 3, Biosense-Webster or Optima/Ensite Velocity, St. Jude Medical, St. Paul, Minnesota, USA). Mean LA pressures were recorded by transducing the LA sheath. Blood was aspirated from the femoral vein, right atrium, LA, and coronary sinus ostium for later analysis. RF energy was then applied to the PV antra according to standard techniques to perform wide-area circumferential ablation in order to achieve PV isolation. In non-paroxysmal AF, linear ablation or substrate – targeted ablation (e.g. of complex fractionated electrograms) were carried out at the operator’s discretion. Successful PV isolation was confirmed in all patients by demonstration of exit and entry block.

Raw EP mapping data were exported from the system according to the manufacturer’s instructions, and re-formatted to allow 3D geometry and voltage maps to be re-created in analysis software (Paraview). This allowed voltage values to be digitally analysed according to previously published methods [13]; The PV, LA appendage and mitral valve were excluded from analysis. Bipolar voltage of less than 0.5 mV was considered to represent fibrosis, and this was expressed as a percentage of the overall LA endocardial area, excluding the PVs, mitral valve and LA appendage.

Intra-cardiac and peripheral blood aspirated during ablation procedures was analysed using commercially available enzyme-linked immunosorbent assay (ELISA) kits: PIIINP (Elabscience, Beijing, China), gal-3 (Elabscience, Beijing, China) and ICTP (Cusabio Life Science, Wuhan, China). Further details of the ELISA analysis have been previously published [11]. ICTP levels were analysed from coronary sinus blood, gal-3 and PIIINP levels were analysed as a mean of peripheral and intra-cardiac levels, based on the findings of this previous work [11].

All patients were followed up for 365 days according to standard care, with investigation of possible recurrence based on symptoms. In patients without symptoms or documented arrhythmia recurrence after this 365-day period, 24-h electrocardiogram (ECG) monitoring was performed. Arrhythmia recurrence was defined as any documented AF or atrial arrhythmia lasting more than 30 s, occurring more than 60 days after ablation.

### Statistical analysis

Normally distributed data are expressed as mean ± standard deviation. Non-parametric data are expressed as median (interquartile range). Categorical data are expressed as frequency (percentage). Data were assessed for normality using the Shapiro-Wilk test and non-parametric data were log-transformed prior to analysis if possible. For comparison, we separated patients into two groups with above and below median LV ECV values. Differences in characteristics between these groups were then assessed using independent-sample t-tests for continuous variables or chi-squared tests for categorical variables. Where transformation of non-parametric data was not possible, Mann-Whitney U test was performed to compare distributions. Univariate linear regression analysis was performed to examine relationships between LV ECV and baseline characteristics. For the multivariable analysis, the forced-entry model was used to identify predictors. Analysis was carried out using SPSS (version 22, Statistical Package for the Social Sciences (SSPS), International Business Machines, Inc., Armonk, New York, USA). A 2-sided *P*-value of < 0.05 was considered to indicate statistical significance.

## Results

### Patient characteristics

All 31 recruited had CMR assessment. The participants were typical of AF ablation patients and had few comorbidities apart from hypertension (Table 1). Mean LA volume of the cohort was elevated. All patients had LV ejection fraction (LVEF) more than 45%. The majority (80.6%) had paroxysmal AF (PAF), and the remainder had either persistent or long-standing persistent AF, grouped together for analysis as ‘non-PAF’.

**Table 1:** Participant characteristics

Characteristic	Distribution
Age (years)	56.7 ± 12.7
BMI (kg/m^2^)	27.5 (5.9)
Sex	
Female	9 (29.0)
Male	22 (71.0)
AF classification	
Paroxysmal	25 (80.6)
Non-paroxysmal	6 (19.4)
Time since AF diagnosis (months)	51.3 (53.9)
In AF during CMR scan	9 (29.0)
Diabetes mellitus	1 (3.2)
Ischaemic heart disease	2 (6.5)
Hypertension	9 (29.0)
Systolic blood pressure (mmHg)	125 ± 23
Diastolic Blood pressure (mmHg)	78 ± 15
LA Volume (ml)	102 ± 40 minimum 47.5, maximum 195.4
LV end-diastolic volume (ml)	158 ± 37
LV stroke volume (ml)	93 ± 24
Cardiac output (L/min)	6.3 ± 1.6
LV ejection fraction (%)	59.2 ± 7.1
LV Mass (g)	87.3 ± 24.7
Native T1 (ms)	985 ± 35.5
LV ECV (%)	24.1 ± 2.5
Mean LA pressure (mmHg)	9.0 (5.0)
LA low voltage area (%)	20.5 ± 7.1
ICTP (ng/ml)	317.1 ± 190.1
Gal-3 (ng/ml)	36.4 ± 32.9
PIIINP (pg/ml)	76.4 ± 46.4

*AF* Atrial fibrillation, *BMI* Body mass index, *ECV* Extracellular volume fraction, *gal-3* Galectin 3, *ICTP* Type I collagen C terminal peptide, *LA* Left atrial, *LV* Left ventricular, *LVEF* Left ventricular ejection fraction, *PIIINP* Type III procollagen N terminal peptide

### Analysis

**Fig. 1:**
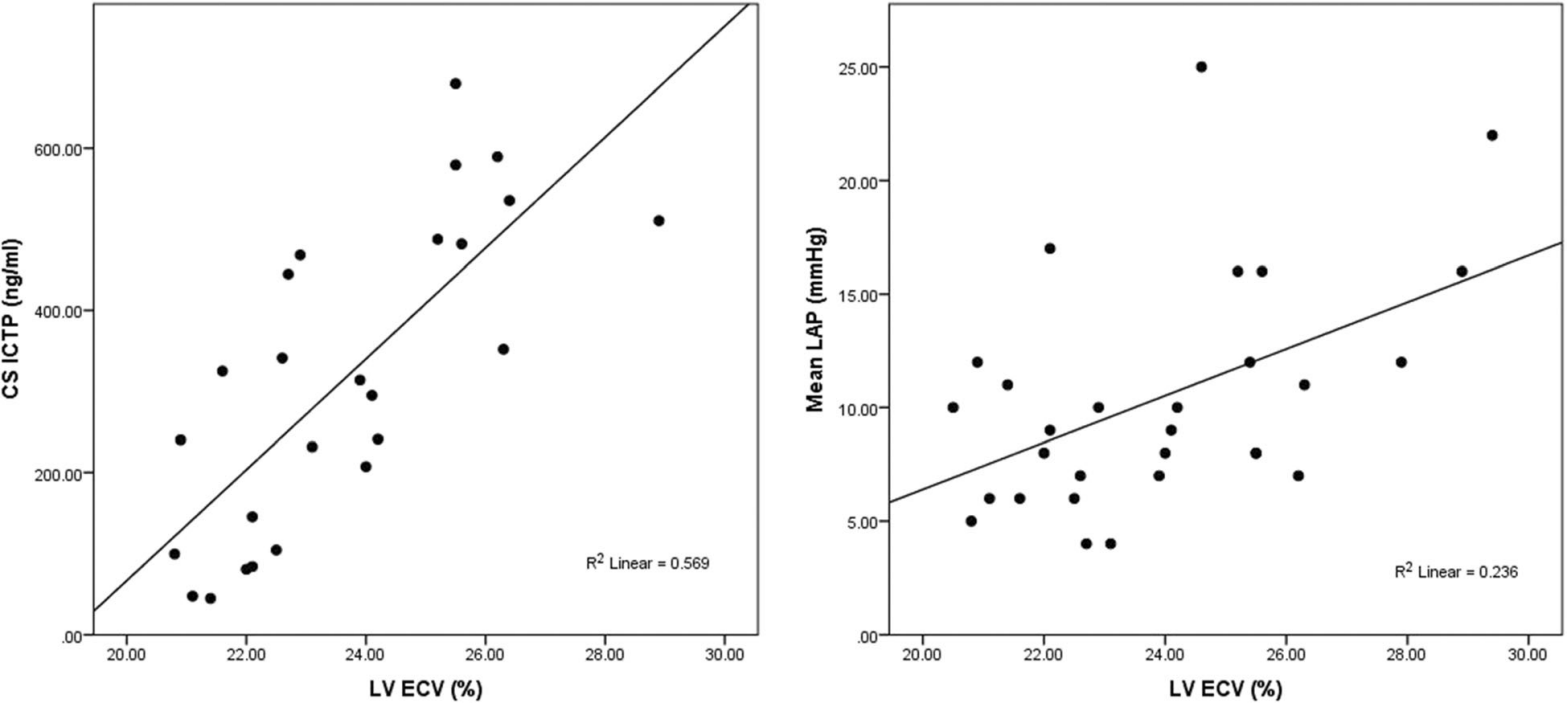
Scatterplots of associations between type I collagen C terminal telopeptide (ICTP)/left ventricular (LV) extracellular volume fraction (ECV) and left atrial (LA) pressure/LV ECV

Table 2 shows the results of the comparisons between the cohort when split above and below the median LV ECV value of 23.9%. The above-median LV ECV group had higher mean LA pressure (13 ± 6 mmHg vs. 8 ± 4 mmHg, *p* = 0.010) and higher ICTP levels (451 (154) ng/ml vs. 212 (146) ng/ml, *p* = 0.001) (Fig. 1). These differences remained significant after multivariable analysis (LAP β = 0.791, *p* < 0.001 and ICTP β = 0.592, *p* = 0.001) (Table 3). The above-median ECV group had a longer duration of AF (52.0 (51.5) months vs 48.8 (57.9) months, *p* = 0.038), but this was not significant on regression analysis. There was no difference in LA low voltage area between the above and below median ECV groups (22 ± 7% vs 17 ± 7%, respectively, 95% CI − 1.06 to 10.9% *p* = 0.102). No other differences regarding CMR assessment were identified, including the other biomarkers.

**Table 2:** LV ECV comparison

	LV ECV		
	Above median LV ECV	Below median LV ECV	*P* Value
ICTP ng/ml	451 (154)	212 (146)	**0.001**
Gal-3 ng/ml	29.2 (19.4)	43.5 (41.8)	0.241
PIIINP pg/ml	63.4 (50.4)	87.2 (58.0)	0.413
LA voltage (% < 0.5 mV)	22.3 ± 7.0	17.4 ± 6.6	0.102
Mean LA pressure (mmHg)	13 ± 6	8 ± 4	**0.010**
Age (years)	56.5 ± 13.0	56.9 ± 12.9	0.918
Time since AF diagnosis (months)	52.0 (31.5)	48.8 (57.9)	**0.038**
BMI (kg/m^2^)	27.4 ± 4.3	29.3 ± 6.0	0.316
LA vol/BSA (ml/m^2^)	46.6 (21.5)	42.9 (15.9)	0.740
LV EDV/BSA (ml/m^2^)	71.4 ± 14.4	79.3 ± 14.9	0.150
LV ejection fraction (%)	61 ± 8	57 ± 6	0.140
LV Mass/BSA (g/m^2^)	43.3 (8.7)	39.1 (10.1)	0.522
Systolic blood pressure (mmHg)	122.8 ± 21.3	127.4 ± 25.8	0.590
Diastolic blood pressure (mmHg)	78.9 ± 12.1	76.3 ± 17.0	0.616
PAF	13 (86.7)	12 (80.0)	0.930
Male	10 (66.7)	12 (80.0)	0.283
Sinus rhythm during scan	11 (73.3)	11 (73.3)	1.00
Recurrence of AF	8 (53.3)	7 (46.7)	0.715

The median value for LV ECV is 24.0%. Values are ‘mean ± standard deviation’, ‘median (interquartile range)’ or ‘frequency (%)’. *P* values represent results of Student’s t-test for normally distributed data, Mann-Whitney U test for non-parametric data, and chi-squared test for categorical data. Statistically significant results highlighted in **bold**. *BSA* Body surface area, *EDV* End-diastolic volume, *PAF* Paroxysmal atrial fibrillation

**Table 3:** Regression analysis

Characteristic	Association with ECV	
	Beta	*P* Value
Age (years)	−0.172	0.354
Time since AF diagnosis (months)	0.038	0.849
BMI (kg/m^2^)	−0.267	0.146
Female sex	0.296	0.106
Non - PAF	0.116	0.535
Hypertension	0.103	0.582
CHA_2_DS_2_VASc	0.008	0.964
LA vol (ml)	−0.247	0.181
LV EDV (ml)	−0.294	0.115
LV ejection fraction (%)	0.184	0.330
LV Mass (g)	−0.250	0.183
Systolic blood pressure (mmHg)	0.033	0.859
Diastolic blood pressure (mmHg)	0.069	0.714
Mean LA pressure (mmHg)	0.486 *0.791*	0.008 ***< 0.001***
% LA voltage (< 0.5 mV)	0.257	0.225
ICTP	0.754 *0.592*	< 0.001 ***0.001***
PIIINP	−0.004	0.989
Gal-3	− 0.142	0.454

Results in *italics* represent multivariable analysis results. Results in **bold** represent statistically significant associations after multivariable analysis

In addition to LV ECV, analysis of native T1 mapping values was performed. An association between with ICTP levels was found on univariable analysis (beta = 0.46, *p* = 0.026). Mean LA pressure also approached significance (beta = 0.348, *p* = 0.070). After multivariable analysis, the association with ICTP remained significant (beta = 0.44, *p* = 0.043) but the association with mean LA pressure did not.

## Discussion

### Associations with CMR T1 mapping parameters

Recent studies have demonstrated that T1 mapping during AF is not only feasible but can give important clinical information [14, 15]. In this study, the presence of AF during the scan appeared to have no effect on the ECV values.

We have shown that mean LA pressure is associated with LV ECV in AF patients, to our knowledge a novel finding.

LV fibrosis appears to be more pronounced in AF patients than in non-AF controls [16]. A potential mechanistic explanation for this is that LV end-diastolic pressure is elevated in the presence of increased ventricular stiffness and diastolic dysfunction, and this in turn causes an increase in LA pressure, dimension and altered function as a result of the increased atrial workload during ventricular diastole [17]. In their analysis of over 400 patients, Park et al showed that elevated LA pressure is associated with both electro-anatomical remodelling of the LA, and AF recurrence after ablation [10]. It therefore follows that an increase in LV ECV may be related to an increase in LA pressure as seen in our study, and, speculatively, incidence and prognosis of AF.

An association between increasing duration of AF and ECV, and in keeping with this, between persistent AF and increased ECV, would be expected based on previous research [7]. Although there was a higher duration of AF in the above-median ECV group, this association was not shown to be significant after regression analysis. An explanation for this may be that the study by Neilan *et. al, *[7], demonstrating the predictive value of LV ECV for AF recurrence, was much larger (*n* = 145) and better powered to detect subtle associations.

Most LV ECV values recorded in this study were within the normal range; indeed, in comparison with data published from our centre, ECV is equivalent to sedentary healthy controls and lower than ECV derived from cohorts with established myocardial pathology [18–20]. This is likely to be due to the patient group selected for this study; those patients undergoing AF ablation are generally at an early point in the development of their AF, predominantly in paroxysmal rather than persistent or long-standing persistent AF, and have little or no clinically relevant underlying structural heart abnormality. This technique may be able to identify at an early stage in the disease process those patients with a lower chance of rhythm control success when AF has been diagnosed. At least one previous study has suggested this, and further research is required to explore this concept further [7].

The other association with LV ECV and native T1 described in this study is with ICTP levels. To our knowledge, this is a novel finding in both instances. ICTP is a product of the catabolism of type 1 collagen, the most abundant form collagen in the myocardium. Studies examining its predictive value in AF ablation are sparse and heterogeneous, but there has been some suggestion that it predicts AF recurrence after rhythm control intervention [21, 22]. In previous work we have shown that coronary sinus ICTP levels are higher than intra-atrial levels in this AF patient cohort, suggesting that the predominant site of increased type-I collagen turnover is the ventricle [11]. This should be considered when interpreting studies which have examined circulating ICTP levels in the context of AF – the association between ICTP and AF may represent ventricular pathology, not atrial [11, 23, 24]. This association may warrant further study, particularly to ascertain any clinical benefit of using this biomarker in AF recurrence risk stratification, or the identification of patients who may benefit from more extensive LA ablation than pulmonary vein isolation.

### Association with LA low voltage

LA voltage mapping data was used as a surrogate marker of LA fibrosis. Although there was more LA low-voltage area in the above-median ECV group, this difference was not significant. Other studies have found low voltage tissue in the LA to be an independent predictor of AF recurrence [5, 6]. The reason for this discrepancy is not clear, but may be related to the small sample size of this study.

### Limitations

A main limitation of this study is the small number of participants. Nevertheless, the study population is representative of AF ablation patients in general and the multiple modality assessment of fibrosis, coupled with the measurement of LA pressure, is unique and has provided novel insights.

A clearly defined value for ‘fibrotic’ tissue based on histological validation was not used, however the results (particularly the association between ICTP and LV ECV) do imply such a relationship exists. In this study, there was no control group with which to compare ECV values. It should be noted that isolated measurement of LA pressure during an ablation procedure may not reflect chronic load status, however repeated or continuous direct LA pressure monitoring is not feasible and the size of this potential error is unknown.

## Conclusion

Higher LV ECV in AF patients is associated with higher LA pressure and type 1 collagen turnover.

## Data Availability

The dataset used during the current study is available from the corresponding author on reasonable request.

## Abbreviations

AF: Atrial fibrillation
BMI: Body mass index
BSA: Body surface area
CMR: Cardiovascular magnetic resonance
ECG: Electrocardiogram
ECV: Extracellular volume fraction
EDV: End-diastolic volume
eGFR: Estimated glomerular filtration rate
EP: Electrophysiology
Gal-3: Galectin 3
ICTP: Type I collagen C terminal peptide
LA: Left atrium/left atrial
LV: Left ventricle/left ventricular
LVEF: Left ventricular ejection fraction
MOLLI: Modified Look Locker inversion recovery
PAF: Paroxysmal atrial fibrillation
PIIINP: Type III procollagen N terminal peptide
PV: Pulmonary vein
RF: Radiofrequency
